# RETINOBASE: a web database, data mining and analysis platform for gene expression data on retina

**DOI:** 10.1186/1471-2164-9-208

**Published:** 2008-05-05

**Authors:** Ravi Kiran Reddy Kalathur, Nicolas Gagniere, Guillaume Berthommier, Laetitia Poidevin, Wolfgang Raffelsberger, Raymond Ripp, Thierry Léveillard, Olivier Poch

**Affiliations:** 1 Laboratoire de Bioiformatique et de Genomique Integratives, Institut de Génétique et de Biologie Moléculaire et Céllulaire, CNRS/INSERM/ULP, BP 163, 67404 Illkirch Cedex, France; 2 Inserm U592 Universite Pierre et Marie Curie, Laboratoire de Physiopathologie Céllulaire et Moléculaire de la Retine, Hopital Saint-Antoine, Paris, France

## Abstract

**Background:**

The retina is a multi-layered sensory tissue that lines the back of the eye and acts at the interface of input light and visual perception. Its main function is to capture photons and convert them into electrical impulses that travel along the optic nerve to the brain where they are turned into images. It consists of neurons, nourishing blood vessels and different cell types, of which neural cells predominate. Defects in any of these cells can lead to a variety of retinal diseases, including age-related macular degeneration, retinitis pigmentosa, Leber congenital amaurosis and glaucoma. Recent progress in genomics and microarray technology provides extensive opportunities to examine alterations in retinal gene expression profiles during development and diseases. However, there is no specific database that deals with retinal gene expression profiling. In this context we have built RETINOBASE, a dedicated microarray database for retina.

**Description:**

RETINOBASE is a microarray relational database, analysis and visualization system that allows simple yet powerful queries to retrieve information about gene expression in retina. It provides access to gene expression meta-data and offers significant insights into gene networks in retina, resulting in better hypothesis framing for biological problems that can subsequently be tested in the laboratory. Public and proprietary data are automatically analyzed with 3 distinct methods, RMA, dChip and MAS5, then clustered using 2 different K-means and 1 mixture models method. Thus, RETINOBASE provides a framework to compare these methods and to optimize the retinal data analysis. RETINOBASE has three different modules, "Gene Information", "Raw Data System Analysis" and "Fold change system Analysis" that are interconnected in a relational schema, allowing efficient retrieval and cross comparison of data. Currently, RETINOBASE contains datasets from 28 different microarray experiments performed in 5 different model systems: drosophila, zebrafish, rat, mouse and human. The database is supported by a platform that is designed to easily integrate new functionalities and is also frequently updated.

**Conclusion:**

The results obtained from various biological scenarios can be visualized, compared and downloaded. The results of a case study are presented that highlight the utility of RETINOBASE. Overall, RETINOBASE provides efficient access to the global expression profiling of retinal genes from different organisms under various conditions.

## Background

The retina is a thin and highly structured layer of neuronal cells that lines the back of eye. Its main function is to convert light energy into an interpretable signal for cortical cells in the brain. The retina has two components – an inner neurosensory retina and an outer retinal pigment epithelium (RPE), which together form the structural and functional basis for visual perception.

The retina consists of several cell types, of which neural cells predominate. Photoreceptors, bipolar and ganglion cells are three principal neuron cell types whose activity is modulated by other groups of cells, such as horizontal and amacrine cells [1]. Defects in any of the above-mentioned cell types can lead to a variety of retinal diseases, including age-related macular degeneration (AMD), retinitis pigmentosa (RP), Leber congenital amaurosis (LCA) and glaucoma. These diseases may cause partial visual loss or complete blindness, depending on the severity.

The recent progress in genomic approaches has now led to an increase in the number of transgenic and knockout animal models that can be used to investigate the role of specific genes in retinal function and related disorders in humans, e.g., *rd1 *is a mouse model for RP [2], *Nr2e3 *for the Human Enhanced S-cone syndrome (ESCS) [3], *Rds *for macular dystrophy and *RPE65*^-/- ^for LCA [4]. Experimental information from the above mentioned models, combined with high-throughput technologies, has led to an increase in the number of experiments related to retinal gene expression.

The recent development of high-throughput technologies has resulted in an enormous volume of gene expression data. General repositories such as GEO [5] and ArrayExpress [6] operate as central data distribution centres encompassing gene expression data from different organisms and from various conditions. In contrast, resources like CGED [7], SIEGE [8] and GeneAtlas [9] are specialized databases that address specific problems; CGED concentrates on gene expression in various human cancer tissues, SIEGE focuses on epithelial gene expression changes induced by smoking in humans and Gene Atlas provides the expression profiles of genes in various mouse and human tissues.

In order to address specific issues related to retina and to meet the needs of retinal biologists in their analysis of gene expression data, we have developed RETINOBASE, a microarray gene expression database for retina. RETINOBASE combines simplified querying, analysis and data visualization options, plus specifically developed meta analysis tools. The integration of gene expression data from various development stages of wild type retina and from diverse conditions and genetic backgrounds will hopefully, not only increase our understanding of the physiological mechanisms involved in normal retinal tissue, but also facilitate studies of gene expression patterns under diverse conditions. Furthermore, RETINOBASE provides a platform for the comparison of different analysis scenarios based on various normalization methods, such as RMA [10], dChip [11], MAS5 [12], and clustering methods, such as the K-means [13] and mixture models methods [14].

## Construction and content

RETINOBASE uses open-source tools. The website is powered by an Apache web server, PHP and Javascript for dynamic web pages and a PostgreSQL object-relational open source database management system (DBMS) as the back end to store data. The RETINOBASE database schema has been developed using the same philosophy as that used to design BASE [15], with enhancements to accommodate data from different platforms and also complies to the Minimum Information About Microarray Experiment (MIAME) standard [16]. It is based on a well-designed relational schema where "realexp" acts as a central table linking expression data with an experiment, sample and array type. This kind of schema helps the system to manage data efficiently, and increases retrieval speed.

RETINOBASE is designed to store gene expression profiles from microarray experiments. We downloaded all publicly available retina-related expression profiles from Gene Expression Omnibus (GEO) yielding 21 experiments [17-32], GEO datasets (GSE 1816, 4756, 1835, 3791, 2868). In addition, 8 proprietary experiments have been incorporated that can be accessed with permission from the owner of the experiment. These experiments were performed under different conditions, including knockout models, treatments and time series experiments performed on different organisms such as drosophila, zebra fish, rat, mice and human. All experiments have complete data, except for one experiment [19] that has partial data at the level of fold change, due to the unavailability of raw data (.CEL) or signal intensity data. Currently, RETINOBASE contains approximately 27 million gene expression values resulting from 509 hybridizations. In future releases of the database, we plan to include data from other studies associated with retina, including the SAGE [33], datasets from Diehn and coworkers [34] who used cDNA array to study human eye tissues, and/or datasets from Blackshaw and coworkers [35] who used SAGE to study mouse retinal development.

### Gene information

In RETINOBASE, the gene annotation information obtained from Affymetrix [36] is linked to information about genes and loci causing inherited retinal diseases, obtained from the Retinal information network (RETNET) [37]. RETINOBASE also provides information obtained from literature about expression of approximately 200 retinal genes specific to certain types of cell, such as photoreceptors, Muller cells or retinal sphere cells.

### Data information

Raw data was obtained in two different formats, either as .CEL files (20 experiments) or at the level of signal intensities (8 experiments). Data obtained at the level of .CEL files are first analysed with three different normalization programs – RMA [10], dChip [11] and MAS5 [12] and then processed using the R statistical package [38] and Bioconductor [39]; after preprocessing, the resulting background-corrected and normalized signal intensities are automatically uploaded to RETINOBASE using SQL scripts via pgAdminIII.

Identification of control samples in an experiment facilitated incorporation of data at the level of fold change in RETINOBASE. The fold-changes in gene expression were calculated as the ratio between the signal intensities of a given gene in the treated (or knockout) model and the control. In the case of experiments performed in replicate, signal intensities were averaged before calculation of the ratios. All the experiments in RETINOBASE were clustered using 3 independent methods: (i) the density of points clustering (DPC) method [40] which is implemented in the in-house FASABI (Functional And Statistical Analysis of Biological Data) software, (ii) the dot product K-means method [41] used in TM4 Multiexperiment Viewer (MeV) a free, open-source system for microarray data management and analysis [42], (iii) the mixture model method implemented in FASABI. Although cluster analyses often provide useful insights into the data, biological interpretation of the results is recommended, since alternative algorithms generally produce different cluster outputs and no single clustering algorithm is best suited for clustering genes into functional groups for all data sets [43]. We chose the DPC, K-means and mixture models methods because of their robustness in clustering large datasets. Although the K-means method generally requires the user to choose the number of clusters to be calculated, the TMEV system uses figure of merit (FOM) graphs [44] to make an appropriate suggestion. Other clustering algorithms, such as a graph-theoretic approach [45], and a neural network based method SOM [46], as well as different parameter options, will be incorporated in future releases of the database. Storing both the normalized and analyzed data in our relational model allows flexible comparisons across different chips at the level of individual genes.

### Quality control

Quality control reports are generated using affyQCReport – an R package that generates quality control reports for Affymetrix array data [47] and RReportGenerator [48] for all experiments, where .CEL files are available. In addition, we also calculate a coefficient of variation for individual Probe Sets between the replicates, which provides a direct estimate of the quality between replicates.

### Experiment and sample details

**Figure 1:**
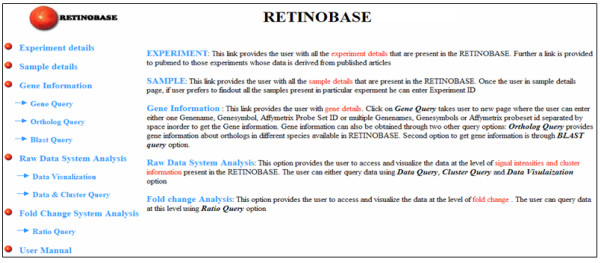
**RETINOBASE home page**. The home page of RETINOBASE [57] which has general information such as experiment and sample details. Specific query options are shown as in the database.

The RETINOBASE home page presents a list of all experiments available to the user and also provides access to experimental details such as title, short description etc. The "Sample details" option (Figure 1) gives details about sample description, organism, tissue, treatment, strain specific information and the array used for hybridisation for a given experiment.

### Querying the database

RETINOBASE has three different querying modules: "Gene Information", "Raw Data System Analysis" and "Fold change system Analysis".

### Gene information module

**Figure 2:**
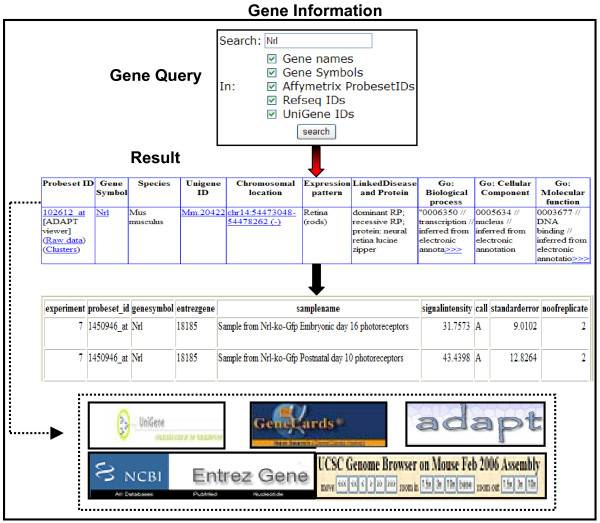
**RETINOBASE Queries**. A "Gene Query" yields information such as Unigene ID, chromosomal location, Entrez gene, expression pattern, linked diseases and gene ontology. The thick black arrow indicates that raw data and cluster information can be accessed directly from a "Gene Query" output, and the dotted line indicates links to external databases.

The "Gene Information" module offers three different query options – "Gene Query", "Ortholog Query" and "Blast Query". Using these, one can access information such as chromosomal location, linked retinal diseases, cellular localization, and gene ontologies for a given gene. Furthermore, gene details returned from these queries are linked to external databases such as GeneCards [49], NCBI [50], specifically to UniGene [51], ADAPT mapping viewer [52] and also to UCSC genome browser [53] that would yield more information (Figure 2).

"Gene Query" and "Ortholog Query" accept as input the gene name, symbol, Affymetrix Probe Set ID, Refseq or Unigene IDs, whereas "Blast Query" accepts sequences in FASTA format. "Ortholog Query" is useful in cross-referencing probe sets between different Affymetrix GeneChip arrays. The data based on reference sequence similarity is taken from HomoloGene and cross-referenced. In addition, the raw data and cluster information for a given gene (cluster number, software used for clustering and information about other genes present in the same cluster) for all experiments can be obtained through the "Gene Query" (Figure 2).

### Raw data system analysis module

**Figure 3:**
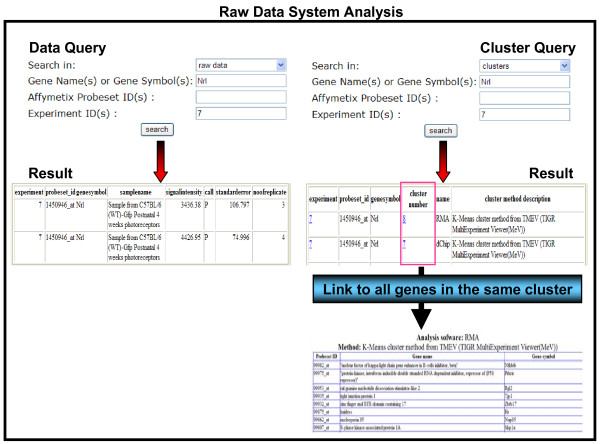
**Data and Cluster Query options**. Data and cluster query results for the *NRL *gene in experiment 7 [17]: "Targeting GFP to new born by NRL promoter and temporal expression profiling of flow-sorted photoreceptors". The user can subsequently obtain all genes present in the given cluster.

**Figure 4:**
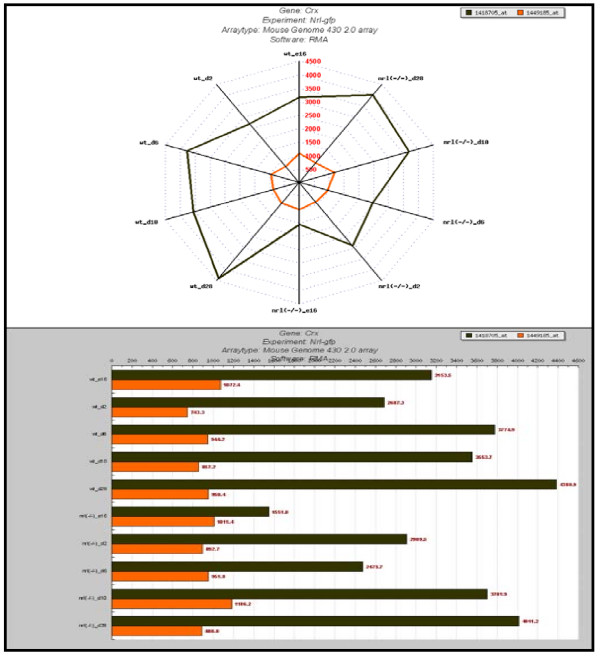
**Data visualization**. Expression profile of two Probe Sets of cone-rod homeobox containing gene (CRX) in the experiment 7 [17]: "Targeting GFP to new born by NRL promoter and temporal expression profiling of flow-sorted photoreceptors". Data is represented as radar plots on the top panel and as histograms in the bottom panel.

This module has "Data and Cluster Query" options and "Data visualization" which is both a query and visualization option. "Data Query" (Figure 3) provides gene expression information at the level of signal intensities for single or multiple genes in all experiments. "Cluster Query" (Figure 3) – unique to RETINOBASE, provides information about expression patterns of related genes across various conditions and genetic backgrounds. It also identifies any two given genes in the same cluster in one or more experiments. Apart from the above mentioned query options, RETINOBASE also provides a user-friendly transcriptomic data visualization tool that was developed to allow retinal biologists to graphically analyse gene expression profiles across all the experiments. A user can choose the experiment, chip, gene and analysis software to be used in a step-by-step process, following which the related samples can be labelled and organized for an easy comparison through histograms or radar-graph representations (Figure 4). This step-by-step process effectively increases querying speed, which in turn allows faster retrieval of specific data from large volumes of gene expression information. Additional information concerning the number of Probe Sets for a gene on a given chip, the normalization software used to obtain the signal intensities and the quality control report of the experiment are also provided.

### Fold change system analysis module

Gene expression information at the level of fold change is provided for single or multiple genes in one or more experiments. In addition, "Ratio Query" supports a specialized query that permits retrieval of all genes from one or more experiments having a fold change greater and/or less than a given criteria.

### Downloading results and user manual

In order to allow users to further compare and interpret data, the results from all querying modules available in RETINOBASE can be downloaded in the comma separated value (.CSV) file format using the "Download results" option.

A user manual is also available on the home page of RETINOBASE and it would provide a detailed description of the utilities.

## Case study: Use of meta-analysis tools in RETINOBASE

**Figure 5:**
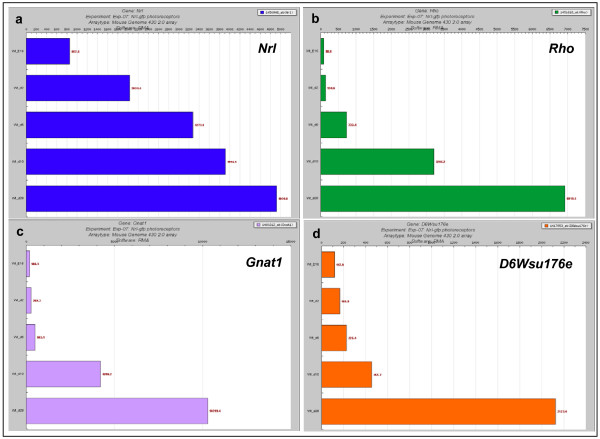
**Expression levels (normalised signal intensities) of *Nrl***. (a) Rho, (b) Gnat1, (c) D6Wsu176e, and (d) at embryonic day 16, post natal day 2, 6, 10 and 28 in experiment 7 [17].

In order to demonstrate the utility of RETINOBASE, we undertook a case study to identify novel genes that may have a potential role in retinal function. In the experiment "Targeting of GFP to newborn rods by Nrl promoter and temporal expression profiling of flow-sorted photoreceptors" (experiment 7 in RETINOBASE) it was elegantly demonstrated that *Nrl *(neural retina leucine zipper) is a key regulator of photoreceptor differentiation in mammals [17]. We first performed cluster analysis using the "Signal intensity or Cluster query" tool in RETINOBASE by providing *Nrl *as the gene symbol and then retrieved the resulting clusters. In agreement with the original study by Akimoto *et al*.,, our "cluster query" found *Rho *(rhodopsin), *Nr2e3 *(nuclear receptor subfamily 2, group E, member 3) and *Pde6b *(phosphodiesterase 6B, cGMP-specific, rod, beta) in the same cluster as *Nrl *in 4 out of 5 possible combinations (1. RMA normalized data and K-means clustering with TMEV, 2. RMA normalized data, K-means clustering with FASABI, 3. dChip normalized data, K-means clustering with TMEV, 4. dChip normalized data, K-means clustering with FASABI and 5. dChip normalized data, clustering with mixture model), confirming that genes specific for rods are coregulated with *Nrl*. In addition, *Gnat1 *(guanine nucleotide binding protein (G protein), alpha transducing activity polypeptide 1), a gene implicated in congenital stationary night blindness [54], was also found in the same cluster in all 5 cluster combinations mentioned above, confirming its role in retinal function. This suggests that *Gnat1 *is also coregulated with *Nrl *in retina. Based on the similar coexpression profiles in wild type mouse retina at time points corresponding to embryonic day 16, post natal day 2, 6, 10 and 28 (Figure 5), we further identified a novel gene that is likely to be implicated in regulating retinal differentiation, namely *D6Wsu176e *(DNA segment, Chr 6, Wayne State University 176, expressed), described as being expressed in the outer nuclear layer of neural retina [55]. The RETINOBASE "Ortholog query" for *D6Wsu176e *points to the human ortholog, *FAM3C*, that is involved in cell differentiation and proliferation during inner ear embryogenesis [56]. With its known function in cell differentiation and its presence in the same cluster as *Nrl *in 3 out of 5 of the above mentioned clustering combinations, *D6Wsu176e *may be an interesting candidate for studying rod differentiation. We further went on to check whether *Nrl*, *Rho, Gnat1 *and *D6Wsu176e *genes are coexpressed (present in the same cluster) in other experiments present in RETINOBASE, in particular checking experiment 12 (Gene expression patterns in the retina of rds mice treated with CNTF/rAAV virus and non-treated after 60 days of injection) (GEO: GSE4756) and experiment 14 (Biological characterization of gene response in Rpe65-/- mouse model of Leber's congenital amaurosis during progression of the disease) [21]. In these two experiments the four genes mentioned above were present in the same cluster indicating that they might be coregulated. This case study illustrates how RETINOBASE facilitates hypothesis testing for the biologist, and demonstrates how to generate novel hypotheses regarding retinal function and finally, how to identify potential novel targets for human retinopathies.

## Future directions

RETINOBASE is under constant development, including addition of new experiments when available. In addition, data from proprietary experiments can be accessed on approval by individual researchers and will be made generally available after publication. Several functional enhancements are also planned for the future. We will continue to refine and update RETINOBASE with respect to data retrieval, mining and visualization options. Direct upload and meta-analysis options will also be provided.

## Conclusion

RETINOBASE has been developed to store, analyse, visualize and compare retinal-related data in order to provide insights into retinal gene expression in various mouse models and other organisms under diverse conditions. Our database, with different types of query options and powerful visualization tools, allows comprehensive analysis of biological mechanisms/pathways of the retina in normal and diseased conditions. We demonstrated by means of a case study how novel genes such as *D6Wsu176e *(which potentially play an important role in retinal differentiation and development) can be identified using the meta analysis tools incorporated in RETINOBASE. With the addition of new experiments the variety of hypothesis testing options will continuously increase, providing biologists with a valuable tool to gain a better understanding of the retina.

## Availability and requirements

The RETINOBASE can be accessed at [57]. All users must register (name and email address) to obtain a username and password.

## Authors' contributions

RK is involved in database design and development, data analysis, design of the user interface and prepared the manuscript. NG, GB and RR developed the web services and database back end. LP is involved in testing various querying tools. WR is involved in data analysis and helped to draft the manuscript. TL participated in the design of the user interface. OP was involved in overall design of the project and in drafting the manuscript.
